# Inaccurate measures of outcomes in the two-sample Mendelian randomization of vitamin D with miscarriage

**DOI:** 10.1093/hropen/hoae025

**Published:** 2024-04-27

**Authors:** Qian Yang, Yangbo Sun, Deborah A Lawlor

**Affiliations:** MRC Integrative Epidemiology Unit, University of Bristol, Bristol, UK; Department of Population Health Sciences, Bristol Medical School, University of Bristol, Bristol, UK; Department of Preventive Medicine, The University of Tennessee Health Science Center, Memphis, TN, USA; MRC Integrative Epidemiology Unit, University of Bristol, Bristol, UK; Department of Population Health Sciences, Bristol Medical School, University of Bristol, Bristol, UK

Sir,

We recently read the article by Zhang *et al.* (2024), entitled ‘No evidence of a causal relationship between miscarriage and 25-hydroxyvitamin D: a Mendelian randomization study’. This two-sample Mendelian randomization (MR) study relied on publicly available genome-wide association studies (GWAS) and considered two outcomes each obtained from different studies: any versus no miscarriage from FinnGen and the number of miscarriages from the UK Biobank (UKB). We have concerns about the reliability of the results, particularly regarding the number of miscarriages.

The authors used summary data from OpenGWAS for the number of miscarriages (https://gwas.mrcieu.ac.uk/datasets/ukb-b-419/). This GWAS was likely run as part of a project that provides summary genome-wide association statistics for all variables available in the UKB. Such summary data can be very useful in MR studies (Richardson *et al.*, 2020; Zheng *et al.*, 2020). However, researchers using these data need to go back to the original sources and check that what they are using really does address the research question of interest. Unfortunately, it appears that this has not been done by Zhang *et al.* A cross check of the number of women included in their analyses (i.e. 78 700 as shown in Supplementary Table S1 of Zhang *et al.*, 2024) with numbers provided on the UKB website and the most recent GWAS of miscarriage using UKB data (Laisk *et al.*, 2020) would have alerted them to their error. Information on miscarriage in the UKB has to be obtained from two questions regarding miscarriage: question 1 ‘Have you ever had any stillbirths, spontaneous miscarriages or terminations?’ (data-field ID 2774), with only women who responded ‘yes’ to question 1 invited to answer further questions including question 2 ‘How many spontaneous miscarriages? (enter 0 if none)’ (data-field ID 3839). It is clear that only question 2 has been considered in generating the GWAS summary statistics used by Zhang *et al.* (2024). As a result, women who responded ‘no’ to question 1 (i.e. those who have not knowingly experienced a miscarriage, stillbirth, or termination (N = ∼95 817 (Borges *et al.*, 2024)) have been excluded.

In the analyses by Zhang *et al.* (2024), women having ‘zero’ miscarriages were actually those who had at least one stillbirth or termination but no miscarriage. As a result, there is major misclassification for the outcome number of miscarriages. Those women currently categorized into ‘zero’ would be expected to have lower 25-hydroxyvitamin D levels than they should have, i.e. true zeros, the majority of those missing would have higher 25-hydroxyvitamin D levels, if the hypothesis that it protects against miscarriage were true. Such a misclassification could bias genetic variant–outcome associations in GWAS and subsequent two-sample MR results towards the null. Furthermore, Zhang *et al.* reported MR estimates of the effect of 25-hydroxyvitamin D on number of miscarriages as ‘Beta’ without any clear units (see Fig. 3 of Zhang *et al.*, 2024). This means that even if the outcome had been correctly specified, it is not at all clear how to interpret the results from a clinical perspective. ‘Beta’ is commonly used to describe results from linear regression. For example, here, it reflects the mean difference in number of miscarriages per unit increase in the exposure. Irrespective of whether a result in a paper is considered null or not, clear units of the effect are essential.

A related issue is whether women never being pregnant should be included in the MR analyses. Zhang *et al.* (2024) used summary data from GWAS of any miscarriage in FinnGen (Kurki *et al.*, 2023) to avoid sample overlap with the UKB GWAS from which they selected genetic instruments for 25-hydroxyvitamin D. In FinnGen, GWAS of pregnancy and perinatal disorders, including miscarriage, are in all women, meaning that those who have never been pregnant, and therefore could not experience a miscarriage, are included as controls (Yang *et al.*, 2022). It is not possible without individual participant data to exclude those women from analyses. In contrast, access to UKB individual participant data is possible, and researchers can then do MR analyses restricted to women who have ever been pregnant (Yang *et al.*, 2022). We would have expected the authors to at least discuss the inclusion of women who had never been pregnant and what impacts that might have on the interpretation of their MR results.

Recent developments of large GWAS databases, e.g. OpenGWAS (Hemani *et al.*, 2018) and GWAS Catalog (Sollis *et al.*, 2023), make it convenient to identify relevant summary data for two-sample MR analyses. However, there is increasing evidence that this convenience can result in questionable research that, in turn, can adversely affect public health (Carter *et al.*, 2023; Sorjonen and Melin, 2024; Xiang *et al.*, 2024). It is essential that authors think deeply and carefully about their research questions, the GWAS and MR methods they are using, and to check original data sources to ensure their results are unbiased and correctly interpreted. Journal editors and reviewers might also benefit from being particularly alert to MR studies using GWAS summary statistics and to have an expectation that authors indicate in their manuscripts what checks have been done to ensure that the GWAS data is testing what they imply it is testing.
